# Mechanisms of Foreign Body Giant Cell Formation in Response to Implantable Biomaterials

**DOI:** 10.3390/polym15051313

**Published:** 2023-03-06

**Authors:** Farshid Eslami-Kaliji, Niloufar Hedayat Nia, Jonathan R. T. Lakey, Alexandra M. Smink, Mohammadreza Mohammadi

**Affiliations:** 1 Department of Animal Biotechnology, Cell Science Research Center, Royan Institute for Biotechnology, ACECR, Isfahan 8159358686, Iran; 2 Department of Public Health, University of California at Irvine, Irvine, CA 92617, USA; 3 Department of Surgery and Biomedical Engineering, University of California Irvine, Orange, CA 92868, USA; 4 Department of Pathology and Medical Biology, University of Groningen, University Medical Center Groningen, 9701 Groningen, The Netherlands; 5 Dale E. and Sarah Ann Fowler School of Engineering, Chapman University, Orange, CA 92866, USA

**Keywords:** macrophages fusion, biomaterials, foreign body giant cells (FBGCs), immune response, mechanotransduction, actin cytoskeleton

## Abstract

Long term function of implantable biomaterials are determined by their integration with the host’s body. Immune reactions against these implants could impair the function and integration of the implants. Some biomaterial-based implants lead to macrophage fusion and the formation of multinucleated giant cells, also known as foreign body giant cells (FBGCs). FBGCs may compromise the biomaterial performance and may lead to implant rejection and adverse events in some cases. Despite their critical role in response to implants, there is a limited understanding of cellular and molecular mechanisms involved in forming FBGCs. Here, we focused on better understanding the steps and mechanisms triggering macrophage fusion and FBGCs formation, specifically in response to biomaterials. These steps included macrophage adhesion to the biomaterial surface, fusion competency, mechanosensing and mechanotransduction-mediated migration, and the final fusion. We also described some of the key biomarkers and biomolecules involved in these steps. Understanding these steps on a molecular level would lead to enhance biomaterials design and improve their function in the context of cell transplantation, tissue engineering, and drug delivery.

## 1. Introduction

Implantable devices are used in a variety of health-related products including drug delivery systems, biosensors, tissue fillers, tissue regeneration, and cell transplantation. However, the interaction between devices and tissue environment triggers sequential physiological events and a foreign body response (FBR) [1,2]. Depending on the nature of the implant, FBR is generally initiated by an inflammatory response and host tissue reconstruction, which proceeds with the resolution of inflammation as well as wound healing. In some cases, the inflammatory response continues to later stages and leads to chronic inflammation, the formation of foreign body giant cells (FBGCs), and development of a fibrous capsule, which could compromise the functional integration of the implants, in some cases [3,4,5].

**Figure 1:**
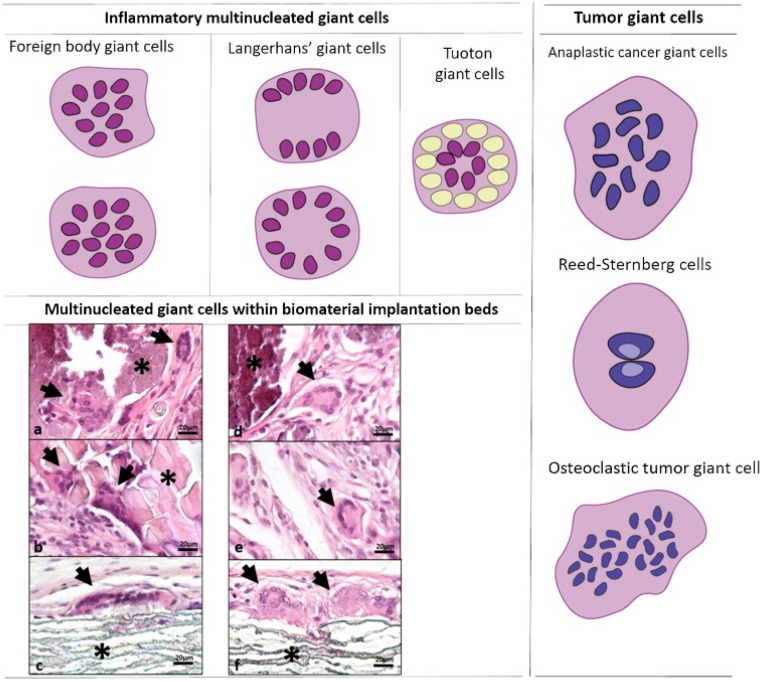
**Schematic representation of the histopathological features of multinucleated giant cell subtypes.** Examples of histological giant cells micrographs are provided, representing the cellular response within the subcutaneous implantation sites of biomaterials. (**a**) Foreign body giant cells with heterogeneously distributed nuclei (black arrows) within the implantation site of a bone replacement material (*) on day 15. (**b**) Foreign body giant cells with heterogeneously distributed nuclei (black arrows) within the implantation site of silk fibroin (*) on day 60. (**c**) Foreign body giant cells with heterogeneously distributed nuclei (black arrows) within the implantation bed of expanded polytetrafluoroethylene (*) on day 60. (**d**) Langerhans’-like giant cells with peripherally oriented nuclei in a circle (black arrow) within the implantation site of a bone replacement material (*) on day 10. (**e**) Langerhans’-like giant cells with peripherally oriented nuclei (black arrow) within the implantation bed of silk fibroin (*) on day 15. (**f**) Langerhans’-like MNGCs with peripherally oriented nuclei in a circle (black arrow) within the implantation bed of expanded polytetrafluoroethylene (*) on day 30. All histological stains are hematoxylin and eosin with 400× magnification. Reed–Sternberg cells are found in malignant tumors, such as Hodgkin’s lymphoma, and have two nuclei. In addition, giant cell bone tumors have evenly distributed nuclei within the cytoplasm. The figure is adapted under creative common license from reference [6].

FBGCs belong to a family of multinucleated giant cells (MNGCs) and can typically be distinguished from other giant cells by pathological examinations. MNGCs include different sub-classes which originate from different progenitor cells, implantation sites, and fusion processes. These subclasses include Touton, Langerhans, osteoclasts, and cancer-related MNGCs (Figure 1). A class of MNGCs called osteoclasts work to remove minerals and bone matrix to promote bone remodeling and regeneration [6]. Langerhans giant cells are formed in response to microbial infections; they contain nuclei that are orientated peripherally and surround the Golgi apparatus [6,7]. Interestingly, formation of Langerhans MNGCs is associated with loss of phagocytosis ability, while preserving the antigen presentability [8]. Xanthogranulomas, fat necrosis, and dermatofibroma are the most common lesions where Touton large cells are found [9]. A ring of nuclei in the center of Touton large cells is surrounded by cytoplasmic lipid buildup [10]. 

FBGCs are known to be the result of macrophage–macrophage fusion, where they fuse in response to particles larger than themselves. While smaller particles could be efficiently cleared by phagocytes, FBGCs are formed where phagocytosis is an insufficient primary mechanism of material degradation. Even though this topic is critical in many biological systems, the mechanistic understanding of the steps leading to macrophage fusion in response to biomaterials is not well understood. More importantly, the function of therapeutic devices may be compromised by FBGCs formation, as they are associated with the fibrous encapsulation and physical walling off around the implant, which enhances material degradation, prevents appropriate molecular transport and vascularization, and remains an internal wound until being removed [6]. We have previously studied the effect of implantation surgical procedure, implant size, implantation location, physicochemical properties of implant surface such as hydrophobicity/hydrophilicity, functional group, protein adhesion, and surface mechanical properties actively involved in FBGCs formation [2,5,11]. However, a mechanistic understanding of FBGCs formation is crucial to develop therapies that may delay or prevent potential adverse events.

In this review, we discuss the cellular and molecular mechanism of FBGCs formation. We discuss steps required for macrophages fusion and FBGCs formation including the development of macrophages fusion competency, actin cytoskeleton rearrangement, macrophages migration, clustering/dispersion of membrane lipid rafts, and cytoplasmic sharing. In particular, we highlight actin cytoskeleton rearrangement contribution in cell protrusions and Mechanosensing/mechanotransduction mediated cell migration. We then discuss the process of clustering/dispersion of membrane lipid raft prior to macrophages fusion. Finally, we briefly summarize some reported consequences of FBGCs formation in the clinical settings.

## 2. Macrophages Fusion Competency

Prior to fusion, macrophages become fusion-competent through the expression of fusogens, facilitating cells migration and adhesion. Fusogens reduce the energy barrier between membranes, which leads to cell–cell attraction despite the electrostatic repulsion dictated by the cell surface. Fusion competency is regulated by the exogenous and endogenous stimuli, which will be discussed below [12].

### 2.1. Exogenous Stimulus for Macrophage Fusion

IL-4, IL-13, and α-tocopherol are examples of exogenous stimuli, which regulate the fusion competency of macrophages [12]. Mclnnis and Rennick’s showed that IL-4-treated mouse bone marrow macrophages achieved fusion competency and formed FBGCs [13]. Translating this finding to human derived macrophages, Mclnnis and Rennick demonstrated IL-4 cannot act as a fusion factor for human macrophages, likely due to the role that IL-4 plays in limiting macrophage adhesion to the plate in vitro [12,14,15,16]. However, pretreatment of macrophages with GM-CSF allows the IL-4 to mediate as a fusion factor [17]. Interestingly, when macrophages were cultured on the RGD-modified polystyrene well plates, adding only IL-4 was sufficient for FBGCs formation [18]. IL-13 induces macrophages fusion and has a unique capacity to induce FBGCs formation in vivo and in vitro [19,20]. Secreted by multiple immune cells, IL-4 and IL-13 lead to the expression of fusogen markers such as dendritic cell-specific transmembrane protein (DC-STAMP) and E-cadherin via STAT6-mediated signaling pathway [19,21]. These observations suggest that both mechanical and biomolecule signaling are required for an efficient fusion competency of macrophages.

α-tocopherol is another exogenous factor to increase FBGCs formation [22]. Schubert et al. added α-tocopherol to the poly (ether urethane urea) elastomers and subcutaneously implanted them into female Sprague Dawley rats. Their results showed that α-tocopherol increased FBGCs formation in vivo, compared to control implants [23]. In vitro studies also showed that α-tocopherol increases macrophage fusion and IL-4-induced FBGCs formation, although this exogenous factor is less potent than IL-4 and IL-13 for FBGCs formation [24,25]. Mechanistically, α-tocopherol activates diacylglycerol kinase, phosphorylating diacylglycerol to produce phosphatidic acid. Phosphatidic acid then induces actin polymerization which allows substantial cytoskeletal rearrangements and cytoplasmic spreading [24]. Overall, IL-4, IL-13, and α-tocopherol are exogenous stimuli that prime macrophages to be prepared for the fusion.

### 2.2. Endogenous Stimulus for Macrophage Fusion

DNAX-activating protein 12 (DAP12) is involved in the regulation of macrophage fusion. DAP12 is a hematopoietic cell surface molecule, and the phosphorylation of its ITAM’s domain (immunoreceptor tyrosine-based activation motif) leads to the activation of phosphatidylinositol 3-kinase (PI3K) mediated by tyrosine kinase Syk [26]. PI3K then regulates the machinery of the actin cytoskeleton through Focal Adhesion Kinase (FAK)-mediated pathways. PI3K is also actively involved in cell survival and cytokine production through extracellular signal-regulated kinase 1/2 (ERK1/2) and NF-κB signaling pathways [27,28,29,30,31]. Helming et al. showed that, by using DAP12 knockdown mice and DAP12 loss of function mice (DAP12 with a nonfunctional ITAM motif), macrophages fusion and FBGCs formation was reduced. On the other hand, overexpression of DAP12 increased macrophages fusion and FBGCs formation in vitro and in vivo. They showed that, in the absence of DAP12 transcript and expression of two fusogen factors, DC-STAMP and Cadherin 1, are at lower levels. Moreover, in both DAP12 knockdown mice and DAP12 loss of function mice, IL-4-induced macrophage fusion was reduced, suggesting that DAP12 is likely independent of exogenous stimuli. Given these results, DAP12 is actively involved in the expression of fusogen markers, and, subsequently, in inducing fusion-competent state [26].

## 3. Cell Migration on Biomaterial’s Surface

After the completion of fusion competency steps, macrophages migrate towards the target and/or each other to be able to fuse. Adhesion of macrophages to the biomaterial surface mediates cell anchoring and activates intracellular signaling pathways that are involved in the development of cell morphology and migration [5,32,33,34]. In the context of biomaterials, protein adsorption on the biomaterials surface support macrophage adhesion, which is likely to contribute to the haptotaxis of macrophages towards each other and FBGCs formation on biomaterials surface [5,35]. Hence, biomaterials that do not support macrophage adhesion and morphological development of macrophages might prevent FBGCs formation. 

### 3.1. Cell Adhesion on Biomaterial

Plasma proteins are adsorbed on the surface of the biomaterial upon implantation, which is mainly regulated by the physicochemical properties of the biomaterial including hydrophilicity/hydrophobicity, charge, roughness, surface chemistry, and surface area [36]. Protein adsorption leads to the formation of Biomaterial Associated Molecular Patterns (BAMP), inducing specific BAMP-associated immune response [37,38,39,40]. Once biomaterials are implanted, recruited monocytes are likely to differentiate to the inflammatory M1 macrophages, possibly due to the presence of IFN-γ, TNF-α, and danger-associated molecular patterns (DAMPs) at the implantation site [41,42,43]. Initial adhesion of macrophages to the adsorbed proteins is mediated through CD11b (a β_2_ integrin) [11], whereas subsequent macrophages adhesion and fusion is mainly triggered by β_1_ integrin. Lv et al. showed that β_1_ integrin steer macrophages phenotypes to pro-regenerative M2 through activating PI3K/Akt signaling pathway, and β_2_ integrin steer macrophages phenotypes to M1 phenotype by NF-κB activation. They also showed that adsorbed fibronectin on hydrophilic surfaces interacts with β_1_ integrin and M2 polarization, whereas fibrinogen on hydrophobic surfaces interacts with β_2_ integrin and M1 polarization of macrophages. This difference is believed to be due to the unique conformation on fibronectin on hydrophobic versus hydrophilic surfaces, necessitating the involvement of different receptors on the surface of macrophages. Although a higher amount of fibronectin adsorbed on the hydrophobic surface, the conformational change of the protein did not support macrophage adhesion [44].

Zaveri et al. studied the role of both CD11b and RGD-binding integrin on the macrophage response to microparticles and material larger than micron scale. They used macrophages that were manipulated with two different techniques. In the first approach, macrophages were derived from CD11b knockout (KO) mice. In the second approach, they blocked the RGD-binding integrin on macrophages. Both approaches were hypothesized to increase the functional life of implanted materials by limiting the chronic inflammation and foreign body capsule formation. Their results showed that the absence of CD11b, as well as blocking the RGD-binding integrin, reduced the in vitro phagocytosis of polystyrene microparticles by macrophage. Two weeks after implantation, fibrotic tissue around subcutaneous implants of polyethylene terephthalate was 30% thinner in the CD11b KO mice and 45% thinner in the macrophages with blocked RGD-binding integrins compared to the wild type control [45].

When integrins on macrophages bind to the adsorbed proteins on biomaterials surface (also known as BAMPs), downstream signaling pathways lead to the expression of cell fusion proteins. These signaling pathways switch macrophages to become fusion competent. They also induce actin cytoskeletal rearrangement that leads to macrophage migration on the biomaterial surface towards other macrophages for fusion. Disruption in adhesion signaling results in anoikis, which is a type of apoptosis due to cell detachment from a supportive matrix [46,47].

Once macrophages are fusion-ready, they need energy and force to move. They must generate traction forces against the BAMP to translocate their body and migrate. These forces are known to be generated from actin cytoskeleton. First, macrophages adhere to BAMP through their integrins while possessing a semi-spherical morphology. The cytoplasmic domain of β integrin binds to several proteins, including talin, tensin, α-actinin, filamin, FAK, and paxillin, which mediate integrin connection to the actin filaments [48,49]. Polymerization of the actin filaments leads to the formation of cell’s protrusions including lamellipodia, filopodia, and podosomes that are actively involved in cell migration and spreading by pushing the cell membrane forward, which lead to the morphological development of the cell [49].

### 3.2. Cell Protrusions

**Figure 2:**
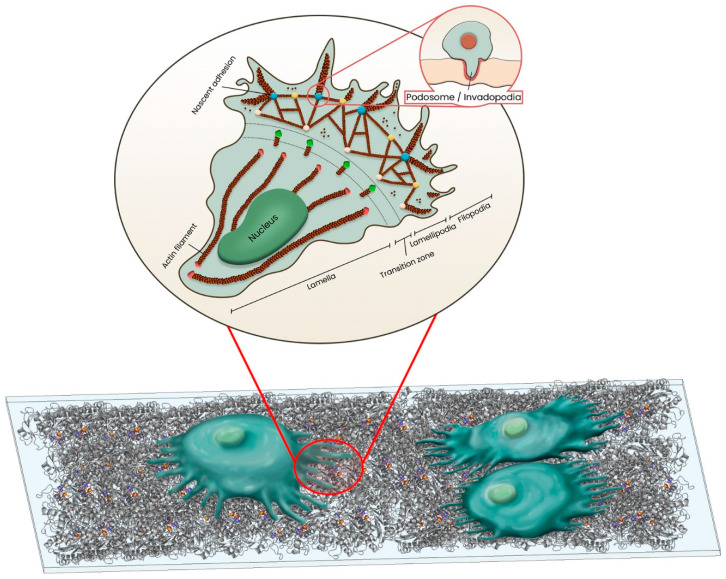
**Actin cytoskeleton architecture of a migrating cell.** Cell migration is associated with the protrusions emerging at the leading edge of the cell called lamellipodia, filopodia, and podosome/invadopodia. However, first, nascent adhesions (blue spheres) are formed in the lamellipodia of motile cells. While the formed diffraction-limited nascent can either disassemble (white spheres), if the biomaterial surface does not support cell adhesion, or elongated at the lamellipodia–lamella interface, if the biomaterial surface support cell adhesion. Nascent adhesions elongation is associated with the actin filament bundles, stabilizing the adhesion formation through actomyosin-induced contractility, and increasing the adhesion size which in turn lead to the adhesion maturation to the focal complex (green spheres), located at the transition zone, and focal adhesion (brown spheres), located at the leading edge of the lamella.

Macrophages migration initiates with formation of small protrusions, which then direct them towards the end location. Lamellipodia are sheet-resembling membrane protrusions, originating at the edge of a spreading and/or migrating cell. Lamellipodia are composed mostly of dendritically branching actin filaments and extend 0.1–0.2 µm from the projecting edge. Lamellipodia serve as the primary locus of cell movement because they include molecular machinery that regulates the arrangement of actin filaments and polymerization/depolymerization (Figure 2) [50,51,52]. 

To get through a thick barrier of ECM, macrophages and dendritic cells require podosomes for chemotactic migration and ECM remodeling. Podosomes are dot-shaped adhesion complexes forming at the cell-ECM interface. Actin-related proteins 2 and 3 (Arp 2/3), the Wiskott–Aldrich syndrome protein (WASP), and cortactin, which are necessary for the control and nucleation of actin, are found in abundance in the core of podosomes.

A ring of actin-associated proteins, including talin, α-actinin, vinculin, paxillin, and integrin, surrounds the core of podosomes. Actin filaments containing myosin-II connect the ring to the core. Since podosomes are dynamic, different cell types exhibit varied podosome morphologies. [53,54]. Similar in design, invadopodia can project farther into the BAMP/ECM. [51,55,56]. On the ventral cell surface, podosomes are often found clustered below the leading edge of the cell, whereas invadopodia are frequently located underneath the nucleus (Figure 2) [57,58]. 

Cells perceive environmental stimuli with the aid of filopodia and move in the right directions. Lamellipodia have protrusions called flopodia, which resemble fingers and are made of parallel, cross-linked actin filaments (Figure 2) [59]. For the F-actin cytoskeleton to attach directly to the ECM substrate, nascent adhesions arise beneath lamellipodia that are behind the leading edge and/or beneath filopodia [58,60]. They are transitory adhesions that last just briefly, might grow longer, or develop into focal complexes [58,61].

Finally, cells repeatedly bind and unbind to the ECM using their focal complexes to maintain structural stability during migration. The focal complex is positioned at the interface between lamellipodia and lamella and is more stable than nascent adhesion. To stabilize the cell during migration, the focal complex matures into bigger focal adhesions, which are greater in size (about 2–6 µm). The synchronization of focal adhesion construction, maturation, and turnover with protrusion of the leading edge at lamellipodia promotes planar cell movement [62,63,64]. In fact, the focal complex must constantly remodel into the focal adhesion and vice versa for cells to migrate (Figure 2) [65].

#### 3.2.1. Actin Cytoskeleton Rearrangement

**Figure 3:**
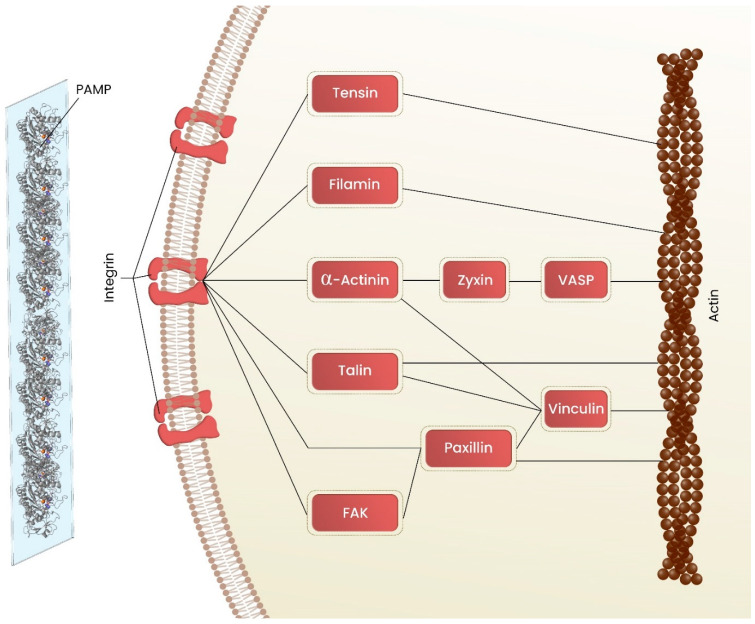
Organization of molecular connections mediating linkages between actin cytoskeleton and transmembrane adhesion receptors, integrins, at the cell–ECM adhesion sites.

The protrusion of adhered cells requires the rearrangement of the actin cytoskeleton to accommodate the restructuring of the macrophages. The polymerization of actin is triggered through agonistic interaction of BAMPs and cell surface integrins (Figure 3).

The binding of integrins to BAMPs connects extracellular substrate to the actin cytoskeleton of macrophage, which creates traction forces. These forces are regulated through integrin-actin cytoskeletal linking proteins including paxillin, talin, a-actinin, vinculin, filamin, etc. Talin and α-actinin bind directly to the cytoplasmic domains of several integrins, while they are also connected to the actin either directly or through vinculin. Talin mediates FAK’s localization to these structures and plays as a key regulator of the inside-out activation of integrins. The α-actinin is associated with force-dependent adhesion strengthening. It has been demonstrated that the knockdown of α-actinin disrupts actin bundles integration and inhibits large and long adhesions formation [65,66].

In order to move forward, cells must first construct nascent adhesions on the lamellipodia, which then develop into focal complexes and grow into focal adhesions in the lamella. The final stage is the breakdown of the rear adhesion, which results in rear-end retraction and dissociation, allowing for cell-body translocation forward. Cell adhesion and migration is a dynamic process due to the rapid formation of nascent adhesions at the leading edge and the disassembly of integrin-mediated adhesions behind the cell [67,68]. Filamin links integrin to the actin cytoskeleton, while preventing integrin activation. Filamin regulates the integrin role in adhesion and dissembling for migration, by either inhibiting talin binding or crosslinking cytoplasmic domains of integrin [69,70,71]. Recent research, however, suggests that it could instead be crucial for immune cell trafficking and adherence to shear flow in vivo [72]. Protrusions-specific components, including paxillin, filamin, talin, and α-actinin, mediate signals that regulate actin arrangement and polymerization through translating mechanical signals into biochemical signals [49,67]. Paxillin and FAK localize 30 nm above the cell membrane, whereas zyxin, vasodilator-stimulated protein (VASP), and α-actinin reside more than 50 nm above the membrane. Talin’s head localize with FAK near the membrane, while its tail localizes with actin [73].

In summary, cell migration is heavily regulated by integrin-ECM interaction. During cell movement, such interactions lead to integrin clustering and the formation of macromolecular complexes. These complexes then control various stages of macrophage movement.

#### 3.2.2. Mechanosensing/Mechanotransduction in Cell Protrusions Mediates the Cell Migration

**Figure 4:**
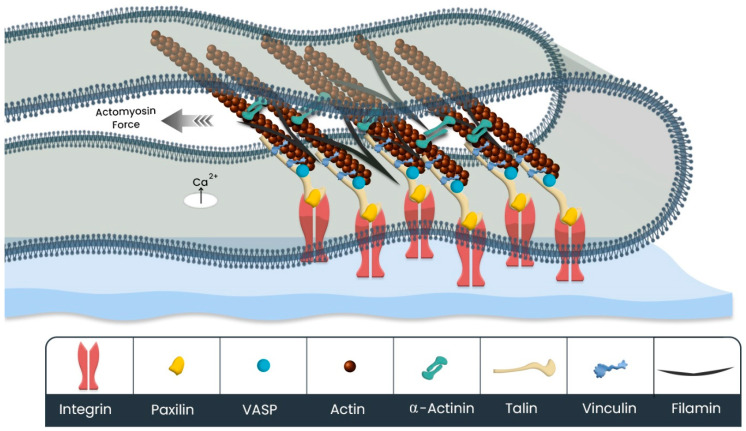
**Schematic of force induction through Myosin II and adhesion maturation.** Actomyosin, which consist of actin filaments and myosin, exerts myosin II-related tension on actin filaments giving rise to backward flow of actin filaments. Then, talin molecules and, subsequently, integrins are stretched, providing binding sites for vinculin. Vinculin binding to both integrin and talin strengthens the linkage between integrin and talin and acts as an anchor for the actin cytoskeleton to the adhesion sites. Additionally, filamin and α-actinin are actively involved in actin-bundling and cross-linking. Meanwhile, new actin monomers are incorporated at the end of pre-existing actin filaments to continue VASP-dependent polymerization of the actin filaments. In the weak adhesions, exerted forces along with actin polymerization lead to the rapid retrograde flow without leading-edge protrusion and transmitting traction force on the ECM. While in the strong adhesion, the generated forces are transmitted to the ECM resulting in leading-edge protrusion and, subsequently, cell mobility. Paxillin, which is co-localized with talin head, is phosphorylated during high traction forces. Ca^2+^ channel is actively involved in creating fusion pore.

When the leading edge of a migrating cell moves forward, nascent adhesion can either grow and elongate or disassemble. Disassembling of nascent adhesion requires actin depolymerization at the lamellipodia–lamella interface. Although nascent adhesion is known to be a weak adhesion, it can mature into a focal complex. Adhesion maturation and elongation is initiated by the formation of short actin bundles from polymerized actin filaments (Figure 4) [74,75]. Actin filaments are crosslinked by α-actinin to form short actin bundles at the front moving edge. These actin bundles are connected to the actin network that contains active myosin II in the lamella. Myosin II generates contractile forces, leading bundles to move rearward. This process gives rise to the bundle elongation and, subsequently, nascent maturation into the focal complex (Figure 4) [61,75,76]. Adhesion maturation is accompanied by significant recruitment of focal adhesion proteins, including vinculin, α-actinin, vasp, and zyxin, to strengthen the link between ECM, integri, and actin. The linkage between the actin cytoskeleton and BAMP-adhered integrin is mediated by talin, without myosin II involvement. This weak link could undergo slippage by forces around 2 pN, leading to the dissembling of nascent adhesion. To strengthen this link, talin expresses vinculin binding site. Vinculin acts as an anchor by binding to both talin and actin. Therefore, the ECM-integrin-actin linkage is strengthened by more than 10 times [50,77,78]. Actin filaments are also polymerized at focal adhesion, where VASP protein promotes actin polymerization. Note that VASP directly binds to the actin cytoskeleton, where Zyxin bridges VASP and α-actinin (Figure 4) [79,80]. The generated forces lead to rearward flow of the actin network, which then converts into the forward movement of the cell. A single integrin molecule at focal adhesions bears between 1 and 40 pN of tensions [50].

As mentioned above, formation of focal adhesions is a critical step determining the migration of macrophages. Keselowsky et al. studied the effect of biomaterial surface chemistry on the formation of focal adhesion. They showed that α_5_β_1_ integrin mostly adhere to the adsorbed fibronectin on the surface with −OH functional group, while α_v_β_3_ showed higher affinity to −COOH functional group. Interestingly, the hydrophilic −OH functional group led to the accumulation of adhesion regulator proteins including talin, paxillin, and α-actinin at the adhesion sites. This accumulation indicates a strong actin-integrin-ECM linkage to support cell migration. In contrast, hydrophobic −CH_3_ substrate led to the lowest level of recruitment of these components [37].

In summary, proteins that mediate the connection between integrin and actin are able to combine biochemical and mechanical cues and transmit this information via biochemical signaling cascades and the mechanical arrangement of the cytoskeleton.

## 4. Cell Migration and Fusion

Upon migration of cells towards biomaterials, they are subjected to the next phase migration towards each other prior to their fusion. Chemokines such as CCL2, CCL4, CCL13, and CCL22 are secreted by adhered macrophages to the BAMPs, leading to the recruitment and migration of other macrophages towards the adhered macrophages. Fusion-competent macrophages chemotactically get into the vicinity of each other [35]. Although the fusion process for the formation of FBGCs has not been well identified yet, three steps have been discovered as requirements for macrophage fusion. These steps are expression of fusogen markers, actin cytoskeleton rearrangement, and quick clustering/dispersion of membrane lipid rafts [6,53,81].

Fusogen markers, particularly M-cadherin, are accumulated in the cell–cell contact region, at the leading edge of lamellipodia where lipid rafts are clustered. The clustering of lipid rafts gives rise to the localization of fusogen markers at the possible fusion region [82]. Although adhesion molecules are necessarily required for fusion, the complex of adhesion molecules impairs the closure of opposed cell membranes; therefore, there is a dynamic cycle between clustering and dispersion of membrane lipid rafts. Lipid raft clustering leads to the accumulation of adhesion molecules at the presumptive fusion site, which is required for the tethering of adhesion molecules. While the dispersion of a lipid raft impairs the localization of adhesion molecules at the presumptive fusion site and leads to homogenous expression of them across the plasma membrane, it has been shown that, by administration of methyl-β-cyclodextrin (MCD), a cholesterol-binding agent, cholesterol was removed from the plasma membrane and, subsequently, lipid rafts were disrupted. Therefore, fusogenic marker localization was also disrupted and lead to the homogenous expression of them across the plasma membrane. Hence, lipid raft is considered a prerequisite for fusion [83].

Mukai et al. proved that the membrane cholesterol concentration is reduced before membranes merge, as increasing the membrane fluidity is required for membranes union. Their results showed that lamellipodia move quickly, until after the initial contact which is mostly formed by E-cadherin, while lipid rafts cycle between clustering and dispersion. Then, lamellipodia stop moving and lipid rafts are clustered at the leading edge; here, the membrane adheres to the adjacent cell. Fusogenic markers are possibly engaged at this step, as lipid rafts are then laterally dispersed from the lamellipodia center, prior to the membranes merging. Finally, membranes merge takes place at the lipid raft-free region [83].

However, there are contradictory reports about the initial contacts. While some studies reported that initial contact is formed by E-cadherin-enriched filipodia [6,81], Balabiyev et al. showed that nectin-2 and E-cadherin-enriched podosomes induce membranes adhesion. They showed that a zipper-like structure (ZLS), a transient dynamic structure with a 13 min lifespan, is formed through podosomes. The podosome transition into the ZLS is induced by E-cadherin and nectin-2, through the bridging of the membranes. It seems that ZLSs are assembled in a sequential manner and in one direction, zippering plasma membranes [53].

Prior to cytoplasmic sharing through fusogen markers ligation, lipid raft clustering and disrupting is required for overcoming electrostatic repulsion of cells membrane. Within milliseconds, lipid raft clustering and disrupting facilitates the contact of two membranes through binding of their fusogens. Upon accomplishing the ligation of fusogens, cytoplasmic sharing initiates, allowing >2 macrophages to fuse into a new cell, known as FBGC.

### Fusogens Are Involved in Macrophage Fusion

When two macrophages are in the vicinity of each other, E-cadherin adheres to actin cytoskeletons of two cells, forming initial contacts. There are several proteins mediating actin cytoskeleton linkage with E-cadherin. β-catenin is the most important one, connecting to the α-catenin, which is required for actin filaments bundling and, subsequently, reinforcing the cell–cell adhesion [84,85]. The cell–cell initial contact through E-cadherin also triggers Rho-family GTPases signaling pathways, leading to reorganization of actin cytoskeleton. The activity of Rho family gives rise to the myosin II activation as well as inhibition of myosin light chain kinase (MLCK). The activation of myosin II is associated with generating contraction forces, and inhibition of MLCK delays the expansion of cell–cell contact [86]. During E-cadherin mediated cell–cell adhesion, actin filaments nucleation and polymerization are mediated by recruiting Arp 2/3 and cortactin [53,66].

Fusogenic proteins include DC-STAMP, CD206, and the macrophage fusion receptor. DC-STAMP is a transmembrane protein that increases FBGCs formation both in vitro and in vivo. It has been revealed that cytokines such as IL-13 and IL-4 increase DC-STAMP expression. However, a ligand for this receptor has not been identified. Macrophages fusion was disrupted in DC-STAMP-deficient macrophages [87,88]. 

Lectin receptor CD206 (a mannose receptor) is a critical fusogen for FBGCs formation [89]. The mannose receptor-induced macrophage fusion mechanism has not been identified; however, time-lapse microscopic showed that lack of mannose receptor in myoblasts disrupted chemotaxis capability. Additionally, myoblasts without mannose receptors could not uptake collagen for remodeling, while ECM remodeling is required for migration and accomplished by podosome and/or invadopodia [90,91]. It has been demonstrated that the inhibition of CD206 reduced FBGCs formation both in vitro and in vivo. Both IL-4 and IL-13 upregulate the expression of mannose receptor CD206 [92].

Macrophage fusion receptor is another fusogen that belongs to the family of signal regulatory proteins (SIRPs). Macrophage fusion receptor, also known as SIRP-α, interacts with CD47, which belongs to the Ig superfamily [93,94,95,96]. This interaction not only leads to the fusion of macrophages but also downregulates phagocytosis. Therefore, it can be hypothesized that macrophages lose their phagocytosis ability during the fusion process [94].

Ca^2+^ and soluble NSF attachment protein receptors (SNAREs) are also fusion modulators. SNAREs are broken into target membrane proteins (t-SNAREs) and secretory vesicle-associated proteins, (v-SNAREs). Ca^2+^ bridges between phosphate groups of opposing bilayers. SNAREs bind to the calcium ions, self-assembling into a ring conformation to form a fusion pore [97].

Metalloproteinase MMP9 is also required for macrophage fusion. In contrast, inhibition of the tetraspanins CD9 and CD81 increased macrophage fusion. It has been recently demonstrated that lack of CD9 and CD81 upregulates MMP9 [98,99,100,101]. 

Macrophage fusion is regulated by fusogenic proteins including DC-STAMP, CD206, macrophage fusion receptor, and some metaloproteases. While we know macrophage fusion initiates the cytoplasmic sharing, further investigation is required to reveal downstream signaling pathways that are activated upon ligation.

## 5. Consequences of Macrophages Fusion

FBGCs secrete high levels of cytokines including TGFβ within the biomaterial microenvironment, leading to the transformation of fibroblasts to myofibroblasts. Myofibroblasts may secrete ECM proteins such as collagen, forming a fibrotic capsule surrounding the biomaterial. Collagen deposition is concomitant with neoangiogenesis in the fibrotic tissue. Macrophages and MNGCs, as the sources of VEGF within the tissue, locally secrete VEGF that leads to the initiating of neoangiogenesis [102]. It has been shown that formed neovessels possess irregular-shaped morphology similar to that of tumor vessels supporting metabolic deregulation and tumor progression. However, neoangiogenesis has been proven to significantly contribute to FBGCs formation. Dondossola et al. used the VEGF trap to prevent VEGF availability and, subsequently, inhibit angiogenesis. As a result, angiogenesis has been stopped and led to a significant reduction in collagen deposition and FBGCs formation. Nevertheless, the VEGF trap showed less efficiency in reducing collagen deposition and FBGCs formation compared to macrophages depletion by clodronate liposome. Interestingly, the concurrent application of both methods completely stopped FBGC s and fibrotic capsule formation [103]. It h was proven that even injectable (not implantable) biomaterials can lead to the FBGCs formation in clinical phase. It would be interesting to compare differences between FBGCs in response to implantable versus injectable biomaterials because DAMPs formation is significantly less when biomaterials are injected. The rate of FBGCs formation in four biomaterials used as injectables (Table 1) seems to be lower than implantable biomaterials.

The impact of FBGCs on biomaterials safety and efficiency is not well understood yet. Simplistically, FBGCs may degrade implanted devices through reactive oxygen species (ROS) and degradative enzymes. Furthermore, the rapid release of ROS and respiratory burst leads to cells exhaustion, leading to the loss of cells producing bacteriocidal molecules. Hence, stress cracking and degradation of biomaterials surfaces may give rise to devise failure in some cases, and could ultimately lead to the rejection of implanted devices [4,104].

**Table 1:** Rate of FBGCs formation after biomaterials implantation along with their case studies.

Biomaterial	FBGCs/No of Patients	Ref	Case Report
Polymethylmethacrylate (PMMA) microsphere	15 in 587	[105]	Requena et al. observed Strong FBGCs formation after injection of (PMMA) microsphere in 4 patients. Time of appearance of FBGCs vary between 6 to 14 months [106].
Poly-lactic acid (PLA) microsphere	5 in 722	[107]	PLA-related FBGCs appear 6–24 months after injection [105]
	3 in 2131	[108]	
Poly-hydroxyethyl-methacrylate (pHEMA)	9 in 455	[109]	_
Silicone oil	5 in 608	[110]	Arin MD et al. observed granulomas composed of multinucleated giant cells after 18 months of silicone oil injection in a patient [111].
	1 in 500	[112]	

## 6. Conclusions

Adverse immune responses against biomaterials could significantly impact the safety, accuracy, and/or efficacy of the implants. FBGC formation on the biomaterials surfaces is a critical player to determine the resolution or continuation of the host inflammatory response. Mechanistic understanding of events leading to macrophage fusion and FBGCs formation allows us to better develop targeted therapies to delay, prevent, or resolve adverse inflammatory response to implants. This review elaborately summarized the mechanisms of FBGC formation in a sequential manner. Macrophages become fusion competent through both endogenous and exogenous stimuli. Then, a successful adhesion to the BAMPs leads to the morphological development within macrophages and the emergence of cell protrusions. Macrophages then organize migration through actin cytoskeleton traction forces against formed BAMPs. Finally, fusogens mediate the fusion of macrophages in a dynamic process of clustering and dispersion of membrane lipid raft. We recommend that future research focuses on better understanding the key BAMPs and their characteristics involved in macrophage fusion. We also expect follow-up studies to better demonstrate the functional importance of FBGCs in the context of not only therapeutic but also diagnostic and aesthetic biomaterials.

## Data Availability

Not applicable.
